# Towards developing a consensus assessment framework for global emergency medicine fellowships

**DOI:** 10.1186/s12873-019-0286-6

**Published:** 2019-11-11

**Authors:** Haiko Kurt Jahn, James Kwan, Gerard O’Reilly, Heike Geduld, Katherine Douglass, Andrea Tenner, Lee Wallis, Janis Tupesis, Hani O. Mowafi

**Affiliations:** 1 FRCPCH Belfast Health and Social Trust, Belfast, UK; 2 Friedrich Schiller University, Jena, Germany; 3 FRCEM, FAMS Tan Tock Seng Hospital, Singapore and Yong Loo Lin School of Medicine, National University of Singapore, Singapore, Singapore; 4 MBBS MPH MBiostat Monash University, Melbourne, Australia; 5 MBChB DipPEC MMed Stellenbosch University, Cape Town, South Africa; 6 MPH George Washington University, Washington, DC USA; 7 MPH University of California, San Francisco, CA USA; 8 FCEM(SA), PhD University of Cape Town, Cape Town, South Africa; 9 MD University of Wisconsin, Madison, WI USA; 10 MPH Yale University, New Haven, CT USA

**Keywords:** Global emergency medicine, Global health, Assessment, Curriculum, Evaluation, Medical education, Postgraduate medical education, Fellowships

## Abstract

**Background:**

The number of Global Emergency Medicine (GEM) Fellowship training programs are increasing worldwide. Despite the increasing number of GEM fellowships, there is not an agreed upon approach for assessment of GEM trainees.

**Main body:**

In order to study the lack of standardized assessment in GEM fellowship training, a working group was established between the International EM Fellowship Consortium (IEMFC) and the International Federation for Emergency Medicine (IFEM). A needs assessment survey of IEMFC members and a review were undertaken to identify assessment tools currently in use by GEM fellowship programs; what relevant frameworks exist; and common elements used by programs with a wide diversity of emphases. A consensus framework was developed through iterative working group discussions. Thirty-two of 40 GEM fellowships responded (80% response). There is variability in the use and format of formal assessment between programs. Thirty programs reported training GEM fellows in the last 3 years (94%). Eighteen (56%) reported only informal assessments of trainees. Twenty-seven (84%) reported regular meetings for assessment of trainees. Eleven (34%) reported use of a structured assessment of any sort for GEM fellows and, of these, only 2 (18%) used validated instruments modified from general EM residency assessment tools. Only 3 (27%) programs reported incorporation of formal written feedback from partners in other countries. Using these results along with a review of the available assessment tools in GEM the working group developed a set of principles to guide GEM fellowship assessments along with a sample assessment for use by GEM fellowship programs seeking to create their own customized assessments.

**Conclusion:**

There are currently no widely used assessment frameworks for GEM fellowship training. The working group made recommendations for developing standardized assessments aligned with competencies defined by the programs, that characterize goals and objectives of training, and document progress of trainees towards achieving those goals. Frameworks used should include perspectives of multiple stakeholders including partners in other countries where trainees conduct field work. Future work may evaluate the usability, validity and reliability of assessment frameworks in GEM fellowship training.

## Background

Global Emergency Medicine (GEM) is a subspecialty that sits at the intersection of Global Health (GH) and Emergency Medicine (EM) [1]. The subspecialty developed organically over years and encompasses a wide range of medical and public health activities around the world including: development and implementation of emergency care systems in various settings from low-resource settings to even some high-resource settings that do not yet have formal emergency care; development of EM as a recognized medical specialty where it does not formally exist; health care during complex emergencies; and research to advance the science and practice of emergency care globally [2].

This wide range of GEM activities also encompasses diverse skills – research techniques, project management, logistics, public health training – that are not routinely included in most EM training programs. As such, fellowship programs have been developed for focused mentorship and training of individuals interested in making GEM their career.

GEM training varies widely in duration and structure, ranging from experiences integrated into longer EM residencies (as is common in many places globally where EM residency training is longer) to post-graduate training programs of 1–2 years duration after residency (as is the case in North America where residency training is limited to 3–4 years). Some GEM fellowships incorporate graduate degrees in related sciences (e.g. Public Health, Epidemiology, Education) [3].

While the particular assessment needs of these diverse programs may differ in detail, there is broad agreement among fellowship directors for the need for structured assessment of GEM fellows and fellowships to ensure consistency and quality of both the graduates and programs that trained them. Further, consistent assessment through a recognized framework can better position graduates of GEM fellowships as they pursue careers with international health agencies or academia by providing a common understanding of what has been achieved in GEM fellowship training.

Out of this broad agreement, fellowship directors of the International Emergency Medicine Fellowship Consortium (IEMFC) – a consortium of North America based GEM fellowships – aimed to develop a common framework for assessment of fellowship trainees. There was recognition that while training formats vary between countries, the principles of assessment would be common to GEM programs globally. There was also agreement that such assessment should be developed in harmony with colleagues from around the world engaged in similar training. As a result, a working group was created including IEMFC members along with members of the International Federation for Emergency Medicine (IFEM) Education Committee to jointly develop a common framework for assessment of GEM training.

The purpose of this article is to provide a review of current approaches to assessment currently in use, to consider common elements needed for GEM fellowship assessments, to present examples of how such common elements may be used to develop assessment tools for GEM fellowship programs with different areas of focus, and to present consensus-based recommendations. This paper then goes further to align assessment to core curricular elements for GEM fellowships and link them to resources available in the literature. Finally, assessment in the context of professionalism and social accountability is discussed.

## Consensus process

The IEMFC invited 20 international leaders in GEM to form a working group with the aim of defining core elements of GEM fellowship training. Invited experts were divided into four groups including curriculum, teaching and learning, assessment, and administration. A working group consisting of five members of this expert panel was tasked with proposing an assessment framework for GEM trainees that would: a) incorporate core elements of training; b) assess formal didactic content as well as field-based work; and c) apply to a broad range of program types currently in existence.

A scoping background review of current assessment frameworks for global health trainees was conducted to identify core elements of such frameworks. The review incorporated assessment tools used for both graduate and post-graduate training but was limited to publicly available frameworks in English. In addition, the IFEM assessment framework for specialist training in EM [4, 5] was referenced to guide the development of this GEM fellowship framework.

Further, a brief survey was sent to all current and former IEMFC programs (40 in all) to assess their current method of assessment of trainees. Solicitation for the brief electronic Qualtrics survey (Qualtrics, Provo, UT, USA) was done by email to the last listed fellowship director. A follow-up email was sent to all non-respondents after one week to prompt completion of the survey. The brief 4-question survey identified whether programs had trainees in the last 3 years and how they conducted assessment of trainees. For those who reported formal assessments of GEM fellows a follow-up survey was sent regarding whether they used an established rubric and whether assessment incorporated formal feedback from partners in other countries. (Appendix 1 – IEMFC Survey). Working group members met via videoconference quarterly over one year to discuss the findings from the scoping review, brief surveys and to discuss elements of proposed assessment framework. The results of the review and survey were combined with experience of the working group members as GEM fellowship directors and educators to generate a proposed assessment framework. (Table 1 – Sample Assessment Framework for Research Based GEM Fellowship).

**Table 1:** Sample Assessment Framework for Research Focused GEM Fellowship

Goals		Outcomes	Output	Activities	Indicators	Means of Verification	Risks/Assumptions
Overall		Attain proficiency in designing, implementing, and publishing GEM research	GEM Specialist prepared to conduct independent GEM research	GEM Fellowship training	Completion of GEM Fellowship activities	Certification by GEM Fellowship Director	N/A
Professionalism	1	Develop skills for stakeholder engagement	Develops stakeholder team for project during training	1. Conduct stakeholder analysis for project	Completion of stakeholder analysis	Written stakeholder analysis submitted to fellowship director	N/A
	2	Develop skills in organization and implementation of GEM projects	Competency in GEM project implementation	1. Participate in existing GEM project 2. Design GEM project for implementation during or after fellowship	1. Participation as measured by fellowship director 2. Completion of project design	1. Certification of fellowship director 2. Submission of written project design	GEM Fellow has adequate time to complete project during training
	3	Develop cooperative relationships with existing medical, public health and governmental organizations	Develop professional and mentorship network for future GEM work	1. Identify mentor in area of interest 2. Attend professional conferences/meetings 3. Engage in working groups for development of GEM	1. Identification of mentor(s) 2. Attend at least one professional conference per year during fellowship 3. Participate in at least one GEM working group during fellowship	1. Submit list of actual or potential mentors in area of interest to fellowship director 2. Certificate / CME from professional conference 3. Submission of working group output or summary of activities to fellowship director	Funds available for attending conferences annually
Communication	4	Develop educational skills and presentation techniques	Competence in delivering educational lectures and scientific presentations	1. Deliver educational lectures in medical / clinical setting 2. Deliver oral presentations as part of formal degree or certificate programs 3. Deliver scientific presentation at conference or department	1. Evaluation of educational / scientific presentations in medical settings 2. Evaluation of educational/scientific presentations in academic setting (e.g. as part of MPH or other formal degree or certificate training) 3. Letters certifying performance from GEM partners in-country or from organizations to which presentation was given	1. Review of written evaluations of presentations given 2. Review of assessments / grades from formal degree or certificate programs 3. Review of letters of performance from partners	GEM Fellow gives educational or scientific presentations as part of training
	5	Develop skills in communication with health authorities	Competence in written and oral communications with leadership of organizations	1. Involve fellows in program leadership meetings with increasing level of responsibility	1. Fellow to take lead on at least one oral and one written communication with project / program leadership	1. Review of sample written communications between fellow and leadership team 2. Direct observation of fellow communications with partners	GEM Fellow project involves field research
Medical Knowledge & Patient Care	6	Develop skills for managing EM patients in austere settings	Competence in management of EM patients in various settings	1. Provide direct emergency care in international setting w/ different language or resource level from home institution 2. Take part in simulation of management of common GEM cases 3. Provide direct patient care in complex emergency setting 2. Complete short-course training in management of common GEM conditions	1. Direct observation of fellow providing patient care in GEM setting 2. Evaluation of simulation/ debrief 3. Direct observation of fellow performance in complex emergency setting 2. Evaluation/ Certificate of Completion of Short Course Training	1. Either certification by Fellowship Director or review of evaluations submitted by faculty or partner organization 2. Review of simulation debrief 3. Either certification by Fellowship Director or review of evaluations submitted by faculty or partner organization 3. Submission of certificate of completion of short course	GEM Fellowship incorporates direct clinical care
	7	Develop knowledge of care protocols for EM conditions in different settings	Demonstrate familiarity with published guideline for GEM care for key conditions	1. Review of published guidelines for emergency triage (WHO, ICRC, SATS) 2. Review WHO Emergency Care checklists 3. Review of published global guidelines for management of acute illness and injury for children and adults (ETAT, IMAI, EmOC, etc.)	1. Able to verbalize principles of triage and perform standardised EM triage 2. Able to verbalize principles of checklists and implement in patient encounter 3. Completion of in-person or virtual training courses	1. Direct observation by faculty or member of partner organization 4. Review of grades / certificate of completion of training courses	N/A
	8	Acquire knowledge of major global health conditions and GEM care	Demonstrate knowledge of top 10 causes of morbidity and mortality globally and which of these are affected by emergency care	1. Complete short-course or self-review of top causes of mortality and DALYs and review of priority setting literature (e.g. DCP3) 2. List and prioritize emergency health conditions in local setting 4. Complete formal course work in Global Health	1. Verbalize understanding of major causes of global morbidity and mortality and how these relate to emergency care 4. Performance in formal courses	1. Certification by Fellowship Director 5. Review of transcripts from completed courses	N/A
	9	Use available data for EM system evaluation	Competence in EM system evaluation	1. Complete evaluation of single site using standard assessment tool (e.g. WHO IMEESC [6], ESRA T[7]) 5. Conduct/Participate in WHO Emergency Care Systems Analysis (local, regional or national)	1. Produce report on emergency care capacity at single site 5. Completion of ECSA report	6. Submission of completed reports to Fellowship Director	GEM Fellowship incorporates field experience in emergency care setting
	10	Develop skills in EM quality improvement	Competence in designing and implementing QA project	1. Complete one quality assurance project at partner site (may involve analysis of existing data) 2. Produce report of QA analysis 6. Communicate findings to partner institution	6. Completion of QA project report	7. Submission of QA report to Fellowship Director	GEM Fellowship provides sufficient time to conduct QA project and to reflect changes
Research Skills	11	Develop skills to critically review GEM literature	Competence in evaluating published GEM literature	1. Conduct peer-review for (1) GEM article per year 3. Lead (1) journal club on GEM topic	1. Completion of peer-review report 7. Journal club activity	1. Review of at least one peer-review report with Fellowship Director or other faculty 8. Evaluation of journal club presentation by Fellowship Director or other faculty	GEM Fellow gets assigned peer-review by journal
	12	Develop skills to obtain funding for GEM research	Funding proposal for GEM research during / after GEM fellowship	1. Attend funding / development proposal workshop 2. Identify potential funding agencies 3. Identify call for proposal or open-call in area of interest 4. Develop draft funding proposal	1. Meet with Fellowship Director or other GEM faculty to review funding agencies and calls for proposals 8. Completion of draft funding proposal	1. Submission of draft funding proposal to Fellowship Director 2. Submission of proposal to funding agency 9. Funded proposal	Sufficient funding available to attend workshop
	13	Acquire understanding of research methodology	Competence in selection of proper study methods for GEM research	1. Complete course in research methodology – overview (online, self-study, or class) 5. Complete training in *specific* research methods (online, self-study, or class)	9. Certificate of completion or transcript	10. Review of transcripts / certificates by Fellowship Director	Sufficient funding for research methods training. May use free online courses
	14	Develop skills in data management & analysis	Competence in research data management	1. Complete coursework in data science-collection, storage, cleaning and analysis of data. (Online, self-study, or class) 2. Complete coursework or self-study in data visualization (online, self-study or class) 6. Training in literature review	10. Certificate of completion or transcript	11. Review of transcripts / certificates by Fellowship Director	Sufficient funding for data science training. May use free online courses
	15	Develop research design skills to assess EM care/ intervention	Completed research study protocol	1. Develop research question 2. Develop study protocol 7. Submit study protocol for ethics review	1. Clearly-stated hypothesis 2. Completion of study protocol ready for ethics review 11. Ethics review submission completed	12. Certification by Fellowship Director	N/A
	16	GEM research study implemented	Data gathered and ready for cleaning and analysis	1. Perform local stakeholder analysis and engage partners 2. Select & train study team 3. Gather data and securely store 4. Clean data in preparation for analysis 8. Complete analysis of data	1. Advisory group (incl. FD & partners in-country) 2. Completion of human subjects training by team members 3. Completion study protocol training (led by Fellow) 12. Secure database of study data	1. Submission of names of advisory group and report of meeting 2. Review of human subjects research training certificates 13. Certification by Fellowship Director or partners	N/A
	17	Gain skills for research manuscript preparation and publication	Research manuscript prepared and ready for peer-review	1. Conduct literature review for GEM research project 2. Analyse GEM research data 3. Complete data visualization 9. Prepare manuscript	1. Submission of literature review 2. Completion of data tables 3. Completion of figures 13. Completion of manuscript	1. Certification by Fellowship Director 14. Submission of manuscript	GEM Fellowship sufficient duration to allow completion of research project

## IEMFC survey results

Responses were received from 32 IEMFC programs (80%). All but two programs responded that they had trained fellows in the last 3 years (94%). Not all programs actively recruit trainees each year (on average there are only 15–20 applicants annually for IEMFC fellowships) and programs were instructed to reply if they had trainees within the last 3 years. The majority of programs (27, 84%) used regular meetings to discuss goals, objectives and progress, while 4 (13%) programs indicated assessment by ad hoc meetings with trainees and 1 program (3%) described only summative reports at the end of training. The methods of assessment varied widely with 14 (44%) programs using only oral communications, 7 (22%) using some form of written assessment but no specific instrument, while 11 (34%) programs described some form of a structured tool for assessment. Of those using structured tools, only 2 (18%) utilized validated instruments which were adaptations from general pediatric and EM residency assessment tools for electives. Only 3 (27%) reported incorporating formal written feedback from colleagues and partners in other countries on the performance of GEM fellows in the field.

## Scoping review results

The scoping review yielded several different approaches to assessment used for global health trainees (not specific to EM or for trainees who had already completed EM specialty training.) In order to create a framework relevant to GEM fellowships, it is important to analyse a few of these to identify common elements that may be useful in developing an assessment framework tailored to GEM fellowships.

The Consortium of Universities for Global Health (CUGH) has proposed a framework for Inter-Professional Global Health Competencies [8] that could be adapted by program directors for GEM fellowships (Table 2). It is assumed that those pursuing dedicated training as part of a GEM fellowship will be in Level III or IV of this framework.

**Table 2:** CUGH FRAMEWORK Global Health Competency [8]

Level I: *Global Citizen* For all post-secondary students pursuing any field with bearing on global health. Level II: *Exploratory* For students at an exploratory stage considering future professional pursuits in global health or preparing for a global health field experience working with individuals from diverse cultures and/or socioeconomic groups. Level III: *Basic Operational* For students aiming to spend a moderate amount of time, but not necessarily an entire career, working in the field of global health. Level IIIa: *Practitioner-Oriented Operational* Required of students practicing 1) discipline-specific skills associated with direct application of clinical and clinically-related skills acquired in professional training in one of the traditional health disciplines; and 2) applying discipline-specific skills to global health-relevant work from fields that are outside of the traditional health disciplines (e.g., law, economics, environmental sciences, engineering, anthropology, and others). Level IIIb: *Program-Oriented Operational*: Required of students in the realm of global health program development, planning, coordination, implementation, training, evaluation or policy. Level IV: *Advanced* Level Required of students whose engagement with global health will be significant and sustained. These competencies can be framed to be more discipline-specific or tailored to the job or capacity in which one is working. This level encompasses a range of study programs, from a masters level degree program, up to a doctoral degree with a global health-relevant concentration. Students enrolling in these programs are usually committed to a career in global health-related activities.	

The CUGH framework was further adapted by Douglass et al. to establish global health milestones for learners in Emergency Medicine [9]. Through their work, each of the CUGH domains was further elaborated to detail specific competencies from novice to expert practitioners. This work provides an excellent resource for GEM fellowships to assign expected levels of proficiency for their graduates.

In addition, IFEM has a 10-step assessment framework applied to the overall IFEM core curriculum for general EM training [4, 5] (Table 3). These 10 principles of best practice may also be used to guide the development of assessment strategies in GEM fellowship training as a subspecialty of EM.

**Table 3:** IFEM Curricular Assessment Framework [4]

1) Define the purpose of the assessment 2) Select an overarching competency framework 3) Define progression from novice to expert 4) Design a blueprint of the curriculum 5) Select appropriate assessment methods 6) Decide on the stakes of the assessment 7) Involve stakeholders in the design of the assessment programme 8) Aggregation and triangulation of assessment results 9) Assessor selection and training 10) Quality improvement	

Further, Health Education England [10] has published a toolkit for the appraisal and collection of evidence of knowledge and skills gained through participation in an International Health Project (Table 4). It provides a reflective portfolio from prior to departure until the return to record the fellowship experience. The sections relate directly to the core elements of the NHS Knowledge and Skills Framework, which have been mapped to the domains required for medical revalidation:

- Domain 1 – Knowledge, skills and performance.
- Domain 2 – Quality Assurance.
- Domain 3 – Communications, partnership and teamwork.

**Table 4:** Health Education England Toolkit for the collection of evidence of knowledge and skills gained through participation in an international health project [10]

1) Prior to departure 2) Complete ‘before’ section of self-assessment form 3) Preparation for volunteering 4) Volunteering experience 5) Following return to the UK 6) After appraisal	

This toolkit provides a minimum standard of a portfolio of evidence for appraisal and supervision for any GEM experience and suffice for the assessment of short GEM programs of just a few months without further formal assessment. However, for full-length GEM fellowships (1–2 years), a more extensive assessment related to a curricular framework may be appropriate.

There was broad agreement from the working group members that while programs are by nature very different, all programs should provide knowledge in the field of global public health and program development in addition to field experience. Using the IFEM Framework for Curricular Assessment as a guide we propose an assessment framework for GEM fellowships to guide knowledge acquisition, as well as, professionalism and social accountability in field experiences [3, 4, 11, 12].

### Competency framework & progression from novice to expert

While core curricular elements for GEM curricula have been suggested [2, 13] there is no unified curriculum for such fellowships [14]. The further development and specialization of GEM fellowships over time may lead programs to choose a subset of these suggested elements and expand them in terms of detail and scope within their particular area of focus.

Whether programs choose to take these core curricular elements together or adapt them to create a novel set, they should then apply an assessment framework to them to measure both how trainees are doing in achieving these competencies as well as how the program is doing in delivering the training.

Field work is integral to any GEM fellowship. While the format of field experiences will vary, practical experience in the field implementing lessons learned is fundamental to becoming a GEM professional.

A “tick box” approach to assessment which focuses on task completion rather than attainment of competency does not adequately assess how trainees/fellows actually perform [15]. Increasingly, a “Milestones” approach has been adopted whereby different levels of achievement across several domains are identified and trainees progress is tracked [16]. This process has recently been further elaborated for GEM learners [9] in general but has not yet been applied to GEM fellowships.

*Recommendation - It is recommended that each fellowship program develop a list of core general competencies as well as specific competencies related to the focus of their specific program (*e.g. *research, humanitarian health,* etc.*) and regularly evaluate attainment of these competencies in the assessment of their trainees.*

### Mapping the curriculum

In order to assess trainees’ progress during fellowship training, curricular elements should be mapped to core competencies that they support/promote. In 2015, Kwan et al. [4] conducted a detailed mapping process of the curricular elements and assessment methods for both the Accreditation Council for Graduate Medical Education (ACGME) [17] and the Royal College of Physicians and Surgeons of Canada physician competency framework (CanMEDS) [11] using a log frame approach [18]. While not mandatory, a Logical Framework Approach (log frame) allows aims and objectives of the fellowship to be mapped to defined outcomes, learning activities and assessment (achieving curricular outcomes and competencies), which in turn are mapped to monitoring and evaluation [18].

Curricular outcomes are also mapped to indicators that will enable program directors to design assessment programs matched to the outcomes and standards defined in their curricula.

Curriculum, competencies and assessment tools should align to give a true reflection of the trainees’ performance [4, 19, 20]. The selection of appropriate assessment methods brings its own challenges in the GEM training environment [8, 15, 21–24].

*Recommendation – GEM fellowships should map out their curriculum to logically connected curricular elements, competencies to be achieved, and measures of attainment to demonstrate trainee progression through their training program.*

### Postgraduate academic qualifications

There is wide variation globally in GEM training ranging from short experiences to formal 2-year programs. Most of the longer programs include postgraduate academic qualification (e.g. Master’s in Public Health) as part of the fellowship. Review of courses taken can contribute to the didactic assessment of GEM competencies (e.g. core public health topics in low-income countries, study design, data analysis, monitoring and evaluation, others) [3, 11, 12].

*Recommendation – GEM fellowships that include formal didactic training in the form of degree programs or courses taken should review the syllabi of required courses to map how they help to fulfill training.*

### Assessment methods and stakes of the assessment

Each program will need to decide on how it will implement assessments for GEM fellowship trainees. The results of the IEMFC survey indicate that while the majority of programs report regular meetings with trainees to review their progress through training, only a minority of programs utilize any sort of structured instrument to guide such assessments. The result is lack of clarity as to what trainees have achieved in their training.

*Recommendation: Each GEM fellowship program should use the principles outlined in these common frameworks to develop or adapt an assessment framework that is able to characterize the progress of their trainees through their program and can clarify domains in which the trainee may need additional training to achieve competency before the end of their training.*

### Stakeholder engagement and aggregation of assessment results

Social Accountability of medical schools is defined by the World Health Organization (WHO) as “the obligation to direct their education, research and service activities towards addressing the priority health concerns of the community, the region, and/or the nation they have the mandate to serve” [25]. Integration of social accountability into assessment frameworks for GEM fellowships involves taking into account the priorities of multiple “communities” including: the partner communities where GEM fellowships work in their field experiences, agencies with which GEM professionals work (e.g. international organizations, non-governmental organisation (NGO), ministries), as well as the academic EM community to which many GEM graduates will attach for their professional careers in GEM research and program development. Priority health concerns of each of these communities may be different and each GEM fellowship may tailor the elements of social accountability in their assessments to reflect the type of training they focus on, while maintaining core elements of respect for partner communities and their concerns.

The aim of socially accountable GEM fellowships should be to produce fellows who are able to work effectively with local stakeholders to prioritize and address health concerns. Accountability at the individual level requires a tool to help distinguish between novice and expert practitioners. Assessment of social accountability based on the individual fellows’ activities is a must in any assessment program.

The lack of field assessment, in collaboration with the host organisation in-country, limits social accountability. Frequently, those on the ground are best positioned to comment on a trainee’s performance in that environment. Further, incorporation of local partners in assessment of trainees strengthens partnerships and further promotes ethically balanced program development and joint research.

As part of the design of their assessment framework, GEM fellowships should specifically outline how the perspectives of the various communities they endeavor to serve are incorporated as well as which representatives of those communities would contribute to assessment of trainees.

*Recommendation – Integration of social accountability in GEM training may take place in many ways. GEM fellowships should consider doing so in a cross cutting fashion that integrates various stakeholders’ perspectives in design or review of curricula, prioritizing competencies as well as the evaluation of trainees in the field. An example of mapping out elements of social accountability is illustrated in*

**Table 5:** Social Accountability Framework

		RESPONSIBLE	RESPONSIVE	ACCOUNTABLE
A	Project/ Activities/ Research			
1	Identification of Society Needs	Implicit	Explicit	Anticipatory
2	Community Engagement	Community Orientated	Community Based	Community Partnership
3	Ongoing Evaluation			
	a) Focus	Process/Actions	Outcomes	Impact
	b) Data sources/Assessors	Internal	External	Health Partners
	c) Governance	Internal	Internal and External	Health Partners
4	Outcomes	Development and Promotion	Sustainable change	Mutual Transformation
B	Self-Reflection			
1	Core Values	Responsible	Responsive	Accountable
2	Role/Actions	Good Practitioner	Professional Practitioner	Health system Change Agent
3	Personal Impact	Development and Promotion	Sustainable change	Mutual Transformation

Table 5*.*

### Quality improvement

In addition to providing an objective method of evaluating trainees’ progression through training, assessment frameworks can also provide valuable information to GEM program directors in assessing and improving their training programs. When developed jointly these instruments can provide faculty in GEM programs with insights about content and teaching methods provided in colleagues’ programs and provide opportunities for each program director to round out the educational offerings in their individual program. Further, over time the results of assessments of programs trainees, coupled with other information (e.g. survey of past graduates) can inform a programs curricular development and quality improvement efforts.

*Recommendation: GEM fellowships should share their assessment frameworks and regularly compare them to identify gaps in their training programs offerings. Further, programs should consider implementing periodic surveys of prior graduates to compare their graduates’ impressions of their skills once working as GEM professionals to the results of their assessments during training. Such reality testing will provide important insights regarding the validity of their assessment frameworks over time.*

### Proposal for an assessment framework for GEM fellowship programs

When possible, a valid assessment program should be integrated into curriculum design rather than simply layered on top of a program [19, 26–30]. Assessment can be divided into two primary domains - didactic and fieldwork. GEM fellowships should be able to provide meaningful structured assessments of trainees across both these domains.

It is expected that each fellowship program provides a clearly articulated statement of its goals and competencies to be attained by the end of the fellowship. These outcomes should be mapped to specific competencies which may be derived from other published competencies [2, 13]. An example of such a statement would be: *“We are confident that a fellow completing our fellowship program has attained the knowledge, skills and professional attitudes (competencies) to ….”.*

Using this “mission statement” as a guide, GEM fellowships may either develop their curricula de novo or map their existing curriculum into discrete elements that correspond to specific competencies which trainees should achieve. New programs should design their assessment framework simultaneously while existing programs will necessarily consider their existing curriculum when developing metrics for achievement of stated competencies. (An example framework of curriculum and assessment for research based GEM fellowships is provided in Table 1.)

Most GEM fellowships range from 1 to 2 years and we recommend that trainees be evaluated at least twice annually and ideally quarterly with respect to their achievement of stated competencies. While customized assessment frameworks will serve such programs best, shorter programs, like those integrated into EM postgraduate programs, may readily make use of more general assessment tools like those outlined in the introductio n[9, 10]. Many have been developed and validated for similar experiences and will provide enough structured assessment for these short global health experiences.

*Recommendation:*

*GEM fellowships should use a structured process to define the key elements of training, identify who are their communities of concern, and identify how assessments of trainees will take place and by whom. Recommendations for what such a structured process would like are illustrated in*

**Table 6:** Recommendations for Developing GEM Fellowship Assessment

1) Articulate statement of goals and objectives for fellowship 2) Define list of competencies either de novo or based on previously published competencies for GEM 3) Map competencies to curricular outcomes specific for each GEM fellowship 4) Identify which elements are didactic competencies and which are practical/field based 5) Define your *communities of concern* and the health problems this GEM fellowship will address/focus on 6) Identify core assessors to include members from each community of concern for that GEM fellowship 7) Outline a format and interval at which fellows are assessed 8) Describe mechanism by which the program itself is evaluated including evaluation by fellows and graduates of the fellowship program	

Table 6*.*

## Limitations

Like all consensus processes our method for developing a consensus framework is limited by the experiences and biases of the working group participants. While attempts were made to be inclusive of perspective and frameworks globally, it is possible that the perspectives of North American institutions were more reflected as 50% of the lead authors were from US-based institutions. In addition, the brief survey sent to establish current practices in GEM fellowship assessment was sent only to GEM fellowship programs in North America. It was felt to be logistically impractical to survey all GEM programs that may have some international training component, in addition to concerns regarding the variability of the nature of those programs affecting the results of the brief survey. To mitigate this bias, assessment frameworks from the UK for global in-training and post-graduate placements (e.g. Health Education England) were referenced. Finally, as in all surveys of practice, social desirability bias might lead respondents to report more optimistic reports of the frequency of their assessments than actually take place.

## Conclusions

GEM fellowship programs developed organically over several decades and encompass a variety of different areas of focus. Such programs developed out of a recognized need for specialized skills that were not routinely attained in traditional EM training. The lack of standardized assessment of GEM trainees has been recognized as a limitation to demonstrating the impact of these programs as well as to demonstrating the competency and effectiveness of their graduates. Leaders in GEM training have recognized the importance of developing such assessments as a crucial step in advancing the professionalism of GEM. Despite the diverse emphases of these programs, creation of assessment frameworks is an achievable goal that all programs should incorporate into their training programs. Using the approaches outlined above GEM programs can implement rational assessment of their trainees.

## Data Availability

Any data and materials associated with this manuscript may be obtained by request from HM by email to: hani.mowafi@yale.edu

## Abbreviations

CanMEDS: Royal College of Physicians and Surgeons of Canada Physician Competency Framework
CUGH: Consortium of Universities for Global Health
EM: Emergency Medicine
GEM: Global Emergency Medicine
GH: Global Health
IEMFC: International Emergency Medicine Fellowship Consortium
IFEM: International Federation for Emergency Medicine
UK: United Kingdom
WHO: World Health Organization

## References

[1] Arnold JL, Holliman CJ (2005). Lessons learned from international emergency medicine development. Emergency Medicine Clinics.

[2] Bayram J, Rosborough S, Bartels S (2010). Core curricular elements for fellowship training in international emergency medicine. Acad Emerg Med.

[3] Mitchell R (2015). Training in global emergency care: international experience and potential models for Australasia.

[4] Kwan J, Jouriles N, Singer A (2015). Designing assessment Programmes for the model curriculum for emergency medicine specialists. Cjem..

[5] Hobgood C, Anantharaman V, Bandiera G (2011). International Federation for Emergency Medicine Model Curriculum for emergency medicine specialists. Emergency medicine Australasia : EMA.

[6] World health Organization. Integrated Management for Emergency and Essential Surgical Care (IMEESC) toolkit. World Health Organization. https://www.who.int/surgery/publications/imeesc/en/. Accessed 30 November, 2018.

[7] sidHARTe. Emergency Services Resource Assessment Tool. sidHARTe. https://www.mailman.columbia.edu/research/strengthening-emergency-systems/emergency-services-resource-assessment-tool. Accessed 30 November, 2018.

[8] Jogerst K, Callender B, Adams V (2015). Identifying interprofessional global health competencies for 21st-century health professionals. Ann Glob Health.

[9] Douglass KA, Jacquet GA, Hayward AS (2017). Development of a Global Health milestones tool for learners in emergency medicine: a pilot project. AEM Educ Train.

[10] Longstaff B, Waterfield C (2015). Ritman D, et al.

[11] Frank JR. The CanMEDS 2005 physician competency framework: better standards, better physicians, better care. In:2005.

[12] Dyne PL, Strauss RW, Rinnert S (2002). Systems-based practice: the sixth core competency. Acad Emerg Med Off J Soc Acad Emerg Med.

[13] VanRooyen MJ, Clem KJ, Holliman CJ, Wolfson AB, Green G, Kirsch TD (1999). Proposed fellowship training program in international emergency medicine. Acad Emerg Med.

[14] Jacquet GA, Vu A, Ewen WB (2014). Fellowships in international emergency medicine in the USA: a comparative survey of program directors' and fellows' perspectives on the curriculum. Postgrad Med J.

[15] Eichbaum Q (2015). The problem with competencies in global health education. Academic medicine : journal of the Association of American Medical Colleges.

[16] Beeson M, Christopher T, Heidt J (2016). The emergency medicine milestones project.

[17] Accreditation Council for Graduate Medical Education (ACGME) Core Competencies. http://www.acgme.org/acWebsite/home/home.asp. Published 1999. Accessed 01 Nov, 2018.

[18] Couillard J, Garon S, Riznic J (2009). The logical framework approach–millennium. Proj Manag J.

[19] General Medical Council. (2010). Workplace Based Assessment: A guide for implementation. In:2010.

[20] Cydulka RK, Emerman CL, Jouriles NJ (1996). Evaluation of resident performance and intensive bedside teaching during direct observation. Acad Emerg Med Off J Soc Acad Emerg Med.

[21] Hayden SR, Dufel S, Shih R (2002). Definitions and competencies for practice-based learning and improvement. Acad Emerg Med Off J Soc Acad Emerg Med.

[22] Jouriles N, Burdick W, Hobgood C (2002). Clinical assessment in emergency medicine. Acad Emerg Med Off J Soc Acad Emerg Med.

[23] Larkin GL, Binder L, Houry D, Adams J (2002). Defining and evaluating professionalism: a core competency for graduate emergency medicine education. Acad Emerg Med Off J Soc Acad Emerg Med.

[24] Norcini J, Burch V (2007). Workplace-based assessment as an educational tool: AMEE guide no. 31. Medical teacher.

[25] Boelen C (1995). Prospects for change in medical education in the twenty-first century. Academic medicine : journal of the Association of American Medical Colleges.

[26] Wass V, der Vleuten C, In Y, Carter Y, Jackson N. Assessment in medical education and training 1. In: Medical Education and Training: Oxford University Press; 2008. p. 358.

[27] Schuwirth LWT, Van der Vleuten CPM (2011). Programmatic assessment: from assessment of learning to assessment for learning. Medical teacher..

[28] van der Vleuten CPM, Schuwirth LWT (2005). Assessing professional competence: from methods to programmes. Med Educ.

[29] Van der Vleuten CP, Schuwirth LW, Scheele F, Driessen EW, Hodges B (2010). The assessment of professional competence:building blocks for theory development. Best Pract Res Clin Obstet Gynaecol.

[30] van der Vleuten CPM, Schuwirth LWT, Driessen EW (2012). A model for programmatic assessment fit for purpose. Medical teacher..

